# *Trichinella spiralis* Infecting Wild Boars in West, Southwest, and Northwest of Romania: Evidence of an Underrated Risk

**DOI:** 10.3390/microorganisms12050856

**Published:** 2024-04-25

**Authors:** Ana-Maria Marin, Tudor Rareș Olariu, Dan-Cornel Popovici, Gianluca Marucci, Sorin Morariu, Daian Popa, Narcisa Mederle

**Affiliations:** 1 Department of Parasitology and Parasitic Diseases, University of Life Sciences “King Michael I” from Timisoara, 300645 Timisoara, Romania; anamaria.marin@usvt.ro (A.-M.M.); sorinmorariu@usvt.ro (S.M.); narcisamederle@usvt.ro (N.M.); 2 Department of Infectious Diseases, Center for Diagnosis and Study of Parasitic Diseases, Victor Babes University of Medicine and Pharmacy, 300041 Timisoara, Romania; 3 Forestry Faculty, Transilvania University Brasov, Sirul Beethoven, 500123 Brasov, Romania; danpopovici30@yahoo.com; 4 Department of Infectious Diseases, Istituto Superiore di Sanità, Viale Regina Elena 299, 00161 Rome, Italy; gianluca.marucci@iss.it; 5 Department of Surgery, Emergency Discipline, “Victor Babes” University of Medicine and Pharmacy, 300041 Timisoara, Romania; daian-ionel.popa@umft.ro

**Keywords:** *Trichinella spiralis*, *Sus scrofa*, Romania

## Abstract

The species of the genus *Trichinella* are etiological agents distributed all over the world and are able to infect mammals, birds, and reptiles. *Trichinella spiralis* is the species most adapted to domestic and wild pigs and is also the most important etiological agent of trichinellosis. The wild boar (*Sus scrofa*) is a nocturnal omnivorous mammal belonging to the Suidae family. *S. scrofa* has a great appetite and its diet includes a variety of small prey such as mice, rats, and other rodents, as well as carcasses of larger animals. The aim of this study was the identification and the molecular characterization of *Trichinella* larvae isolated from the muscle tissue of *S. scrofa* specimens collected in different counties of Romania. The muscle samples were examined by artificial digestion and the larvae identified at the species level by multiplex PCR. *T. spiralis*, a species that is able to infect a considerable number of different host species including humans, was identified. In Romania, *S. scrofa* is an important reservoir species for *T. spiralis* and plays an important role in linking the domestic and the wild cycle of *Trichinella*, with serious repercussions for human health.

## 1. Introduction

The *Sus scrofa* is an omnivorous ungulate species largely diffused in the hunting fauna of Romania [1]. Being a species with a high adaptability to various types of habitats, the *S. scrofa* represents, in the big game category, the subject of greatest interest for hunting activities [2]. The analysis of data, provided by the Ministry of Environment, Water, and Forests (MEWF) [3] for the 2014–2023 period, revealed an overpopulation of *S. scrofa* until the year 2020. However, after 2020, the population declined due to the African swine fever (ASF) epidemic that affected Romania.

The observed population value in the 2014–2020 period was more than 2.4 times higher than the optimal population value established for this species for the natural hunting habitat of Romania. Even if in recent years, the herds assessed for this species have considerably decreased under the effect of the African swine fever epidemic, they remain at a value of more than 1.4 times higher than the optimal herd value calculated according to natural conditions of habitat, as shown by the MEWF data [3]. This overpopulation was also probably the cause of the rapid expansion of the African swine fever, which, in turn, generated the decrease in the *S. scrofa* herd by more than 30% in just three years (2020–2023). *Sus scrofa* is a species with a very high natural birth rate (186%) [2], having the ability to restore their flocks in relatively short periods of time. The number of hunted animals fluctuated from 25,750 in 2015 to 50,857 in 2020 and then 13,608 in 2023 [3]. Because of this high hunting activity, a remarkable amount of *S. scrofa* meat and raw material was supplied to the Romanian food industry.

Due to the anatomical, behavioral, and feeding peculiarities, as well as hunting management measures, the *S. scrofa* is a perfect host for the transmission of various parasitic zoonoses, particularly the nematodes belonging to the *Trichinella* genus [4,5]. *Trichinella spiralis* is the species most adapted to domestic and wild pigs, and also to rats, having a cosmopolitan distribution. This species is also the most important etiological agent of human disease [6]. The sylvatic cycle of *T. spiralis* involves wild carnivores which can become an important route of transmission of the infection to the domestic cycle. Residues from pigs infected with *T. spiralis* are the main source of infection for synanthropic animals (rats, horses, stray cats, and dogs) [7,8,9]. *Trichinella spiralis* was identified in *S. scrofa* isolates in Germany, Spain, Poland, Finland, Austria, Bulgaria, France, Hungary, and Lithuania, with this wild animal being considered a reservoir host for the established species of the genus *Trichinella* [10]. The epidemiology of trichinellosis caused by *T. spiralis* is in transition in terms of incidence and sources of infection [11]. The consumption of raw or undercooked *S. scrofa* meat represents the source of infection with *Trichinella* spp. larvae, even when they are present in low numbers [12]. The first episode of trichinellosis in humans in the 2015–2016 period was reported in Serbia as a result of the consumption of *S. scrofa* meat [13]. In Portugal, infections of the hunters who consumed *S. scrofa* meat that were not controlled by trichinoscopy and artificial digestion have been reported [14]. In Romania, infection with the *Trichinella* spp. is still significantly present, affecting several species of domestic and wild animals, omnivores, and carnivores, with different prevalence rates over the years and thus maintaining a close epidemiological relationship between the domestic cycle and the sylvatic outbreak of the zoonotic nematode. In *S. scrofa*, in Romania, the species *T. spiralis* and *T. britovi* have been identified [15,16], with a higher prevalence of the latter [17]. The present study was conducted to assess the current epidemiological situation of the *Trichinella* spp. infection in *S. scrofa* in the western regions of Romania. Therefore, this research was aimed at the identification and molecular characterization of *Trichinella* larvae isolated from the muscles of *S. scrofa* from the west, southwest, and northwest of Romania.

## 2. Materials and Methods

### 2.1. Diagnostic Procedures

**Figure 1:**
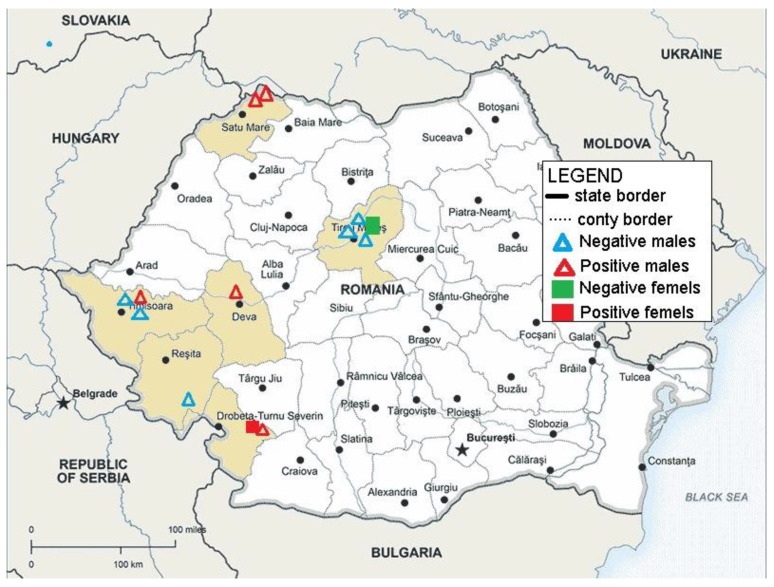
Map showing the geographical areas where the *S. scrofa* carcasses were collected; red triangles show the male-positive sites and the red squares show the female-positive sites.

The research was conducted in the 2021–2022 period, on a total of 20 *S. scrofa* specimens (14 males and 6 females). The *S. scrofa* were shot based on the annual harvest quota approved by the Minister of Environment, Water and Forests. The hunting action by which these *S. scrofa* specimens were harvested was conducted in compliance with Law 407/2006 (on hunting and wildlife protection) [18]. These animals came from different hunting funds, including six counties in Romania (Mehedinți, Caraș-Severin, Timiș, Hunedoara, Satu Mare, and Mureș) (Figure 1).

#### 2.1.1. Artificial Digestion

About 30 g of muscle from the diaphragm of each animal was tested for the presence of the *Trichinella* spp. larvae by the artificial digestion method, according to the Commission Regulation (EC) no. 1375/2015 [19]. After artificial digestion, larvae were collected, counted, stored in 96% ethanol, and sent to the European Reference Laboratory for Parasites (EURLP) (Rome, Italy) for species identification by multiplex PCR [20]. 

#### 2.1.2. PCR Protocol

Briefly, the DNA was purified from single larvae using a DNA IQ System kit (Promega, Madison, WI, USA) and a Tissue and Hair Extraction kit (Promega, Madison, WI, USA). Five primer sets, targeting specific regions (expansion segment V, ITS1 and ITS2) of the ribosomal DNA repeats, were used in multiplex PCR to obtain a species-specific electrophoretic DNA banding pattern [21,22].

## 3. Results

**Figure 2:**
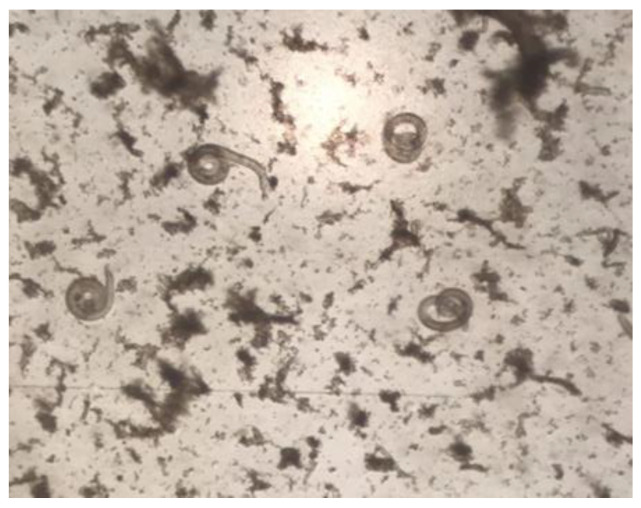
The larvae of *Trichinella* spp. isolated from *S. scrofa* muscle.

Of the 20 specimens, 6 *S. scrofa* from the Mehedinți, Timiș, Hunedoara, and Satu Mare counties tested positive for the presence of *Trichinella* spp. larvae by the artificial digestion method (Figure 2). 

**Figure 3:**
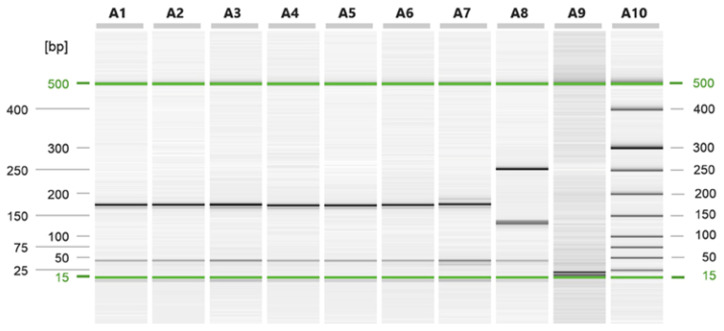
Capillary electrophoresis run of the multiplex PCR on *Trichinella* larvae collected from *S. scrofa*; A1 to A6 larvae isolated from *S. scrofa*; A7 *T. spiralis* positive control; A8 *T. britovi* positive control; A9 negative control; A10 size marker.

Six *Trichinella* larvae have been individually evaluated by multiplex PCR and identified as *T. spiralis* (Figure 3).

## 4. Discussion

In Europe, expanding *S. scrofa* populations represent a potential risk of dissemination of zoonotic nematode infections due to their high susceptibility to infection [11]. Raw or undercooked *S. scrofa* meat and its products are one of the most important sources of human trichinellosis worldwide. In Europe, the following four species of the genus *Trichinella* have been isolated from the muscle of *S. scrofa*: *T. spiralis*, *T. britovi*, *T. pseudospiralis*, and *T. nativa* [23,24]. Although in Eastern Europe, most outbreaks of human trichinellosis are due to the consumption of native pork [25,26], especially those raised in the traditional system, with a substantial risk of infection, nowadays, outbreaks of trichinellosis episodes are associated with the consumption of game meat, especially *S. scrofa* [11].

The epidemiological situation that is registered in Romania is similar to those reported in other European countries, with the species identified in *S. scrofa* being *T. spiralis* and *T. britovi* [16]. The present study revealed a 30% prevalence of the *Trichinella* spp. in *S. scrofa*, in counties from the west, southwest, and northwest of Romania, with the only species identified and molecularly characterized being *T. spiralis*. In the Balkan countries, which are considered endemic for *Trichinella* nematodes in both domestic and wild animals, the prevalence of these zoonotic pathogens in animals is high [27,28]. The dietary habits of consuming raw meat and meat products have led to a remarkably high prevalence of trichinellosis in people living in this European region. Santrac et al. reported the presence of *T. britovi* in *S. scrofa* in Bosnia and Herzegovina [27], and Zivojinovic et al. [28] reported a prevalence of 53% of *T. spiralis* and 31% of *T. britovi* in Serbian wildlife. Hunting in Poland is a longstanding tradition and has become an even more popular phenomenon since 1990, with over 60,000 *S. scrofa* specimens being hunted annually. *T. spiralis* and *T. britovi* were the species isolated from the host muscle [29,30]. A Polish study conducted in 2017 revealed, for the first time, the presence of *T. nativa* (a frost-resistant species) in *S. scrofa*. This finding was not only one of the few cases of *T. nativa* infection in *S. scrofa* worldwide, but also one of the few cases of *T. nativa* detected so far beyond the known range of this species [31]. In Hungary, three species of the zoonotic nematode were identified in *S. scrofa*, namely *T. britovi* (64.7%), *T. spiralis* (29.4%) and *T. pseudospiralis* (5.9%) [32]. Two years later, Tolnai et al. [33] isolated *T. spiralis* and *T. britovi* from the *S. scrofa* muscle and argued that the distribution of *T. spiralis* in Hungarian wildlife was determined by transboundary transmission of the parasite from neighboring endemic countries. The long survival of *T. britovi* larvae was associated with climatic conditions (average annual temperature) that ensured a slower decomposition of wild animal carcasses. In the Iberian Peninsula, *T. britovi* is the representative species in wildlife [14,34], but mixed infection with *T. spiralis* and *T. britovi* was reported in a *S. scrofa* hunted in the province Caceres (Spain) [35]. A mixed infection *T. spiralis/T. pseudospiralis* in *S. scrofa* was also reported for the first time by Nockler et al. in Germany [36]. Langner et al. identified, for the first time, *T. spiralis* in raccoons [37].

An epidemiological study conducted in Italy revealed a stable prevalence of *T. britovi* in *S. scrofa* meat intended for human consumption, suggesting a risk of infection for consumers, especially hunters and users of local markets. The authors recommended that veterinary health education surveillance models be promoted to improve trichinellosis control and prevention in a One Health perspective [38]. The first description in Estonia of *T. psedospiralis* in *S. scrofa* and *T. spiralis* in *S. scrofa* and lynx was made by Karssin et al. [39]. *Trichinella spiralis*, *T. britovi*, *T. pseudospiralis*, and a mixed *T. spiralis/T. britovi* infection were reported in Croatia [40]. The presence of a *Trichinella* infection among wildlife populations suggests a sylvatic cycle of transmission in Argentina, which can serve as a reservoir for humans and domestic animals. The identification of *T. spiralis* species in *S. scrofa* and rats, with a high prevalence in the latter, allowed authors to emphasize the need to improve the pig management process in small individual farms without adequate technology, to improve feed quality, and to upgrade veterinary services to avoid *Trichinella* infection [41,42]. Infections with *Trichinella* spp. have been reported in Vietnam [43]. The predominant species that were isolated from the *S. scrofa* musculature in Israel were *T. spiralis* and *T. britovi* [44], while in Chile, it was *T. spiralis* [45].

The review by Gherman et al. [16] refers to the research carried out over the last 30 years on the presence of *Trichinella* infection in over 80% of Romania’s territory (33 counties). Infections of wild animals were reported in all the studied areas, with the prevalence of *Trichinella* spp. in wild canids and felids being at the top of the epidemiological pyramid, followed by bears, mustelids, and *S. scrofa*. A picture regarding the circulation of *T. spiralis* and *T. britovi* species in Romania was also provided by Nicorescu et al., who isolated *T. britovi* larvae from the muscles of *S. scrofa* and found that they were a higher percentage (57.33%) compared to *T. spiralis* (23.68%). The authors indicated the presence of *T. britovi* throughout the country, especially in areas with high altitudes (hill, mountain), favorable areas for the presence of wild carnivores, which are a reservoir of infection for *S. scrofa*. In the south of Romania, *T. spiralis* predominates in pigs and, implicitly, in the domestic cycle, compared to the north of the country, where the situation is in favor of *T. britovi* [17]. The results of the present study are in line with those previously reported by Blaga et al. [15] and Borza et al. [46]. Comparing to what has been previously observed, this study identified *T. spiralis* as the only species isolated from muscle of *S. scrofa* originating from geographical areas, both with low altitudes (counties Mehedinți, Timiş and Satu-Mare), as well as medium to high altitudes (Hunedoara County). The epidemiological surveillance carried out over a period of six years validated the presence of *T. spiralis* and *T. britovi* in domestic and game animals from the northeastern part of Romania. *T. spiralis* is the dominant species in pigs, horses, *S. scrofa*, and bears and occupies the entire northeastern area of Romania, while *T. britovi* is associated with *S. scrofa* and bears in the mountainous areas [47]. *S. scrofa* meat is a real source of infection with larvae of *Trichinella* spp. even when they are found in sparse numbers [12]. Moreover, the presence of the *Trichinella* nematode in host canid species located in the peripheral habitats of rural localities where traditional hunting activities are frequently encountered may represent a way of transmission of this zoonosis to species of wild fauna of hunting interest (*S. scrofa*) and through it, to the people [16,48,49]. The prevalence of human trichinellosis was described as reaching high percentages in northwestern Romania compared to other regions of the country [50,51]. According to the authors, this could be the result of poor veterinary sanitary control of pork raised in backyards and slaughtered at home, to which culinary habits are added. In this region, hunting practices are common, and pig scraps from backyards and home-slaughtered pigs are left by hunters to attract wild animals [50,51]. Human trichinellosis continues to represent a public health problem in western Romania. Recent studies conducted in this region have shown that the vast majority of trichinellosis cases were severe and patients required hospitalization [25,26]. It should be noted that the number of reported human trichinellosis cases does not illustrate the actual prevalence of the disease. In a seroepidemiological survey conducted in western Romania, anti-*Trichinella* antibodies were detected in healthy blood donors, who were not previously diagnosed with trichinellosis. The demonstration of *Trichinella* antibodies in this group of asymptomatic individuals suggests that in this region, the exposure to the nematode and the prevalence of infection are higher than the number of reported cases [52].

## 5. Conclusions

In the present study, we detected and molecularly identified *T. spiralis* larvae in muscle tissue of *S. scrofa* specimens collected in six different counties of Romania. The animals originated from hunting funds belonging to areas with different geographical features such as low altitudes (Mehedinți, Timiş, and Satu-Mare counties) and medium to high altitudes (Hunedoara County) zones. The *S. scrofa* represents, in Romania, an important reservoir host for *T. spiralis* and a link between the domestic and the sylvatic cycle of this parasite, with an important impact on human health.

## Data Availability

Data are contained within the article.

## References

[1] Micu I., Cotta V., Bodea M. (2008). Vânatul României.

[2] Șelaru N. (2010). Mistrețul.

[3] Ordinul Ministerului Mediului, Apelor și Pădurilor nr. 2847/24.11.2022 Studii de Evaluare a Speciilor De Faună Cinegetică în România. http://www.mmediu.ro/categorie/efective/292.

[4] Brown V.R., Bowen R.A., Bosco-Lauth A.M. (2018). Zoonotic pathogens from feral swine that pose a significant threat to public health. Transbound. Emerg. Dis..

[5] Fredriksson-Ahomaa M. (2019). Wild Boar: A Reservoir of Foodborne Zoonoses. Foodborne Pathog. Dis..

[6] Pozio E., Kapel C.M.O., Gajadhar A.A., Boireau P., Dupouy-Camet J., Gamble H.R. (2006). *Trichinella* in pork: Current knowledge on the suitability of freezing as a public health measure. Euro Surveill..

[7] Dupouy-Camet J., Bruschi F., Dupouy-Camet J., Murrell K.D. (2007). Management and diagnosis of human trichinellosis. FAO/WHO/OIE Guidelines for the Surveillance, Management, Prevention and Control of Trichinellosis.

[8] Oksanen A., Interisano M., Isomursu M., Heikkinen P., Tonanzi D., Oivanen L., Pozio E. (2018). *Trichinella spiralis* prevalence among wildlife of a boreal region rapidly reduced in the absence of spillover from the domestic cycle. Vet. Parasitol..

[9] Pozio E., Marucci G. (2003). *Trichinella*-infected pork products: A dangerous gift. Trends Parasitol..

[10] Pozio E., Rinaldi L., Marucci G., Musella V., Galati F., Cringoli G., Boireau P., La Rosa G. (2009). Hosts and habitats of *Trichinella spiralis* and *Trichinella britovi* in Europe. Int. J. Parasitol..

[11] Murrell K.D. (2016). The dynamics of *Trichinella spiralis* epidemiology: Out to pasture?. Vet Parasitol..

[12] Boros Z., Vallee I., Panait L.C., Gherman C.M., Chevillot A., Boireau P., Cozma V. (2020). Seroprevalance of *Trichinella* spp. in wild boars (*Sus scrofa*) from Bihor county, western Romania. Helminthologia.

[13] Pavic S., Andric A., Sofronic-Milosavljevic L.J., Gnjatovic M., Mitic I., Vasilev S., Sparic R., Pavic A. (2020). *Trichinella britovi* outbreak: Epidemiological, clinical, and biological features. Med. Mal. Infect..

[14] Vieira-Pinto M., Fernandes A.R.G., Santos M.H., Marucci G. (2021). *Trichinella britovi* infection in wild boar in Portugal. Zoonoses Public Health.

[15] Blaga R., Gherman C., Cozma V., Zocevic A., Pozio E., Boireau P. (2009). *Trichinella* species circulating among wild and domestic animals in Romania. Vet. Parasitol..

[16] Gherman C.M., Boros Z., Băieș M.H., Cozma-Petruț A., Cozma V. (2022). A review of *Trichinella* species infection in wild animals in Romania. Food Waterborne Parasitol..

[17] Nicorescu I.M., Ioniță M., Ciupescu L., Buzatu C.V., Tănăsuică R., Mitrea I.L. (2015). New insights into the molecular epidemiology of *Trichinella* infection in domestic pigs, wild boars, and bears in Romania. Vet. Parasitol..

[18] Ordinul Ministrului Mediului, Apelor și Pădurilor nr. 1571/07.06.2022 Privind Aprobarea Cotelor de Recoltă Pentru Unele Specii de Faună de Interes Cinegetic. https://mmediu.ro/articol/ordinul-ministrului-mediului-apelor-si-padurilor-nr-1571.

[19] Commission Implementing Regulation (EU) 2015/1375 of 10 August 2015 Laying Down Specific Rules on Official Controls for *Trichinella* in Meat (Codification). https://eur-lex.europa.eu/legal-content/EN/TXT/?uri=celex%3A32015R1375.

[20] Pozio E., Zarlenga D.S. (2019). International Commission on Trichinellosis: Recommendations for genotyping *Trichinella* muscle stage larvae. Food Waterborne Parasitol..

[21] Pozio E., La Rosa G., Liu D. (2010). Trichinella. Molecular Detection of Foodborne Pathogens.

[22] Zarlenga D.S., Chute M.B., Martin A., Kapel C.M. (1999). A multiplex PCR for unequivocal differentiation of all encapsulated and non-encapsulated genotypes of *Trichinella*. Int. J. Parasitol..

[23] Pozio E., Christensson D., Stéen M., Marucci G., La Rosa G., Bröjer C., Mörner T., Uhlhorn H., Agren E., Hall M. (2004). *Trichinella pseudospiralis* foci in Sweden. Vet. Parasitol..

[24] Pozio E., Kapel C.M.O. (1999). *Trichinella nativa* in sylvatic wild boars. J. Helminthol..

[25] Lupu M.A., Pavel R., Lazureanu V.E., Popovici E.D., Olariu T.R. (2021). Trichinellosis in hospitalized patients from Western Romania: A retrospective study. Exp. Ther. Med..

[26] Pavel R., Ursoniu S., Lupu M.A., Olariu T.R. (2023). Trichinellosis in Hospitalized Children and Adults from Western Romania: A 11-Year Retrospective Study. Life.

[27] Santrac V., Nedic D.N., Maric J., Nikolic S., Stevanovic O., Vasilev S., Cvetkovic J., Sofronic-Milosavljevic L. (2015). The first report of *Trichinella pseudospiralis* presence in domestic swine and *T. britovi* in wild boar in Bosnia and Herzegovina. Acta Parasitol..

[28] Zivojinovic M., Sofronic-Milosavljevic L., Cvetkovic J., Pozio E., Interisano M., Plavsic B., Radojicic S., Kulisic Z. (2013). *Trichinella* infections in different host species of an endemic district of Serbia. Vet. Parasitol..

[29] Bilska-Zając E., Różycki M., Chmurzyńska E., Marucci G., Cencek T., Karamon J., Bocian L. (2013). *Trichinella* species circulating in wild boar (*Sus scrofa*) populations in Poland. Int. J. Parasitol. Parasites Wildl..

[30] Moskwa B., Cybulska A., Kornacka A., Cabaj W., Bień J. (2015). Wild boars meat as a potential source of human trichinellosis in Poland: Current data. Acta Parasitol..

[31] Bilska-Zając E., Różycki M., Chmurzyńska E., Antolak E., Próchniak M., Grądziel-Krukowska K., Karamon J., Sroka J., Zdybel J., Cencek T. (2017). First case of *Trichinella nativa* infection in wild boar in Central Europe-molecular characterization of the parasite. Parasitol. Res..

[32] Széll Z., Marucci G., Ludovisi A., Gómez-Morales M.A., Sréter T., Pozio E. (2012). Spatial distribution of *Trichinella britovi*, *T. spiralis* and *T. pseudospiralis* of domestic pigs and wild boars (*Sus scrofa*) in Hungary. Vet. Parasitol..

[33] Tolnai Z., Széll Z., Marucci G., Pozio E., Sréter T. (2014). Environmental determinants of the spatial distribution of *Trichinella britovi* and *Trichinella spiralis* in Hungary. Vet. Parasitol..

[34] Zamora M.J., Alvarez M., Olmedo J., Blanco M.C., Pozio E. (2015). *Trichinella pseudospiralis* in the Iberian peninsula. Vet. Parasitol..

[35] Rodríguez E., Olmedo J., Ubeira F.M., Blanco C., Gárate T. (2008). Mixed infection, *Trichinella spiralis* and *Trichinella britovi*, in a wild boar hunted in the Province of Cáceres (Spain). Exp. Parasitol..

[36] Nöckler K., Reckinger S., Pozio E. (2006). *Trichinella spiralis* and *Trichinella pseudospiralis* mixed infection in a wild boar (*Sus scrofa*) of Germany. Vet. Parasitol..

[37] Langner T., Hamedy A., Wellner H., Johne A., Mayer-Scholl A., Birka S. (2022). First detection of *Trichinella spiralis* in raccoon (*Procyon lotor*) in Germany. Vet. Parasitol. Reg. Stud. Rep..

[38] Sgroi G., D’Alessio N., Marucci G., Pacifico L., Buono F., Deak G., Anastasio A., Interisano M., Fraulo P., Pesce A. (2023). *Trichinella britovi* in wild boar meat from Italy, 2015–2021: A citizen science approach to surveillance. One Health.

[39] Kärssin A., Hakkinen L., Vilem A., Jokelainen P., Lassen B. (2021). *Trichinella* spp. in wild boars (*Sus scrofa*), brown bears (*Ursus arctos*), eurasian lynxes (*Lynx lynx*) and badgers (*Meles meles*) in Estonia, 2007–2014. Animals.

[40] Balić D., Marucci G., Agicic M., Benic M., Krovina Z., Miskic T., Aladic K., Skrivanko M. (2020). *Trichinella* spp. in wild boar (*Sus scrofa*) populations in Croatia during an eight-year study (2010–2017). One Health.

[41] Cohen M., Costantino S.N., Calcagno M.A., Blanco G.A., Pozio E., Venturiello S.M. (2010). *Trichinella* infection in wild boars (*Sus scrofa*) from a protected area of Argentina and its relationship with the presence of humans. Vet. Parasitol..

[42] Ribicich M., Gamble H.R., Bolpe J., Scialfa E., Krivokapich S., Cardillo N., Betti A., Cambiaggi Holzmann M.L., Pasqualetti M., Fariña F. (2010). *Trichinella* infection in wild animals from endemic regions of Argentina. Parasitol. Res..

[43] Thi N.V., Nguyen V.D., Praet N., Claes L., Gabriël S., Huyen N.T., Dorny P. (2014). *Trichinella* infection in wild boars and synanthropic rats in northwest Vietnam. Vet. Parasitol..

[44] Erster O., Roth A., King R., Markovics A. (2016). Molecular characterization of *Trichinella* species from wild animals in Israel. Vet. Parasitol..

[45] Hidalgo A., Villanueva J., Becerra V., Soriano C., Melo A., Fonseca-Salamanca F. (2019). *Trichinella spiralis* infecting wild boars in Southern Chile: Evidence of an underrated risk. Vector Borne Zoonotic Dis..

[46] Borza C., Neghină A.M., Dumitrașcu V., Tirnea L., Calma C.L., Neghină R. (2012). Epizootiology of trichinellosis in pigs and wild boars in western Romania, 1998–2011. Vect Borne Zoonotic Dis..

[47] Iacob O., Chiruta C., Mares M. (2022). *Trichinella spiralis* and *T. britovi* in North-Eastern Romania: A Six-Year Retrospective Multicentric Survey. Vet. Sci..

[48] Marin A.M., Dărăbuș G., Herman V., Morariu S., Olariu T.R., Dărăbuș R.G., Sîrbu B., Mederle N. (2021). Study on the prevalence and larval burden of the nematode *Trichinella* spp. in red foxes from hunting grounds in Timis county. Rev. Rom. Med. Vet..

[49] Moraru M.M.F., Herman V., Oprescu I., Fodor J.T., Morariu S., Ilie M., Marin A.M., Mederle N. (2022). Wild boar—The source of human contamination with *Trichinella* spp. in Romania. Rev. Rom. Med. Vet..

[50] Blaga R., Durand B., Antoniu S., Gherman C., Crețu C.M., Cozma V., Boireau P. (2007). A dramatic increase in the incidence of human trichinellosis in Romania over the past 25 years: Impact of political changes and regional food habits. Am. J. Trop Med. Hyg..

[51] Neghina R., Neghina A.M., Marincu I., Moldovan R., Iacobiciu I. (2010). Evidence of *Trichinella spiralis* in Timis County, Romania: A Report of a Winter Trichinellosis Outbreak in 2008 Due to Consumption of Contaminated Pork. Vector Borne Zoonotic Dis..

[52] Pavel R., Ursoniu S., Paduraru A.A., Lighezan R., Lupu M.A., Olariu T.R. (2022). Seroprevalence and Risk Factors of Trichinella spiralis Infection in Blood Donors from Western Romania. Medicina.

